# Definition and prevalence of familial short stature

**DOI:** 10.1186/s13052-021-01018-3

**Published:** 2021-03-09

**Authors:** Veronica Grigoletto, Alessandro Agostino Occhipinti, Maria Chiara Pellegrin, Fabio Sirchia, Egidio Barbi, Gianluca Tornese

**Affiliations:** 1 University of Trieste, Trieste, Italy; 2 Institute for Maternal and Child Health - IRCCS “Burlo Garofolo” – Trieste, Via dell’Istria 65/1, 34137 Trieste, Italy

**Keywords:** Familial short stature, Target height, Genetics, Measured, Reported

## Abstract

**Objective:**

To verify the prevalence of novel definitions of familial short stature on a cross-sectional cohort of children referred for short stature when their height and that of both parents were measured.

**Methods:**

We consecutively enrolled 65 individuals referred for short stature when both parents were present. We defined “target height-related short stature” (TH-SS) when child’s height is ≤ − 2 SDS and included in the range of target height; suspected “autosomal dominant short stature” (AD-SS) when child height and at least one parent height are ≤ − 2 SDS; “constitutional familial short stature” (C-FSS) when a child with TH-SS does not have any parents with height ≤ − 2 SDS.

**Results:**

Of 65 children referred for SS, 48 individuals had a height ≤ − 2 SDS. Based on the parents’ measured heights, 24 children had TH-SS, 16 subjects AD-SS, and 12 individuals C-FSS. If we had considered only the parents’ reported height, 3 of 24 children with TH-SS, 9 of 16 with AD-SS, and 10 of 12 with C-FSS would have been lost.

**Conclusion:**

We suggest novel definitions to adequately detect and approach the cases of FSS since C-FSS (25%) might not need any specific investigation, while on the contrary, AD-SS (33%) should undergo genetic evaluation. Moreover, this study underlines that adequate measurement and consideration of children’s and parents’ heights (individually and together) are crucial in the clinical evaluation of every child with short stature.

## Introduction

Short stature (SS) – defined as a height ≤ − 2 standard deviation score (SDS) – is the most common referral reason in pediatric endocrinology [1].

In 23–37% of the cases, these children have a family history of SS and attain a final adult height that, despite being ≤ − 2 SDS, is consistent with their target height (TH): this condition is addressed as familial short stature (FSS) and has long been considered a normal variant of growth and is usually not investigated nor treated [2, 3].

However, genetic analysis’s rapid progress and innovation enabled scientists to identify different monogenic gene defects that cause SS, mainly with an autosomal-dominant inheritance, which may not be classified as FSS according to this definition when the parents have a big difference between their height SDS. For this reason, some authors believe that it is more appropriate to consider FSS when at least one parent has a height of ≤ − 2 SDS [4–6].

To better distinguish a different subset of FSS and their following management, we elaborated the following definitions for FSS:

- **“target height-related short stature” (TH-SS)** when child’s height is ≤ − 2 SDS and included in the range of TH (i.e., TH SDS ± 1.5);
- **suspected “autosomal dominant short stature” (AD-SS)** when child height and at least one parent height are ≤ − 2 SDS, who should undergo genetic evaluation;
- **“constitutional familial short stature” (C-FSS)** when a child with TH-SS does not have any parents with height ≤ − 2 SDS (TH-SS without AD-SS), who does not need further investigations (once that the most frequent causes of SS have been excluded and statural growth has not presented any further deflections over time).

Furthermore, a significant portion of FSS cases may not be recognized in clinical practice if the parents’ height is not adequately investigated. Family history may not be enough to identify all children having a parent with SS if we consider that parents – especially those of children referred for short stature – tend to overestimate their height [7–11].

This study aimed to verify the prevalence of FSS novel definitions on a cross-sectional cohort of children referred for SS when their height and that of both parents were measured.

## Material and methods

We consecutively enrolled 65 individuals referred for SS to the Endocrine Unit of the Institute for Maternal and Child Health “Burlo Garofolo” when both parents were present. After explaining that an accurate estimate of their height was required for their children’s evaluation, parents’ self-reported height was registered (reported parent height, R-PHt). Parents and children heights were recorded to the nearest 0.1 cm (measured parent height, M-PHt, and child height, CHt, respectively) using a Harpenden wall-mounted stadiometer (Holtain 602VR, Crosswell, UK), and children’s weight was digitally registered to the nearest 0.1 kg with an electronic scale (SECA 877, Hmaburg, Germany). Body mass index (BMI) was calculated as weight in kilograms divided by height in meters squared. TH was determined with the formula: (paternal height + maternal height)/2–6 for females and + 6 for males using Growth Calculator 3 Software, and SDS for heights, weights, BMI, and TH, according to Italian reference charts (Cacciari 2006 [12]). Pubertal status was determined following Tanner criteria [13, 14].

All children with SS underwent medical investigations to exclude nutritional, hormonal, and iatrogenic causes for their short stature and were classified according to the definitions mentioned above of FSS (TH-SS; AD-SS; C-FSS).

The study was approved by the Institutional Review Committee (RC 33/18 Line 2).

Data were presented as percentages, median and interquartile ranges (IQRs). Mann-Whitney rank-sum tests and Two-tailed Fisher exact tests were performed to evaluate the relations between variables. A *p*-value < 0.05 was considered statistically significant. Statistical analysis was conducted using JMP™ software (version 15.1.0, SAS Institute Inc.).

## Results

Over 65 children referred for SS, 48 forty-eight individuals had a height ≤ − 2 SDS, with a median age of 9.1 years (81% were pre-pubertal) (Table 1). Although BMI SDS was lower than general population reference (− 1.1 SDS), nutritional, hormonal, and iatrogenic causes have been excluded.

**Table 1:** Data regarding height, weight, and BMI SDS, age, sex, and pubertal stage between groups (data are reported as median and interquartile ranges or frequencies) All data are referred to measured parental heights

	SS (*n* = 48)	No SS (*n* = 17)	***p***	TH-SS (*n* = 24)	No TH-SS (*n* = 24)	***p***	AD-SS (*n* = 12)	No AD-SS (*n* = 36)	***p***	C-FSS (*n* = 12)	No C-FSS (*n* = 36)	***p***
*Height (SDS)*	−2.4 (− 2.8;-2.2)	−1.9 (− 2.0;-1.8)	**< 0.01**	− 2.3 (− 2.5;-2.2)	−2.6 (− 3.0;-2.3)	**0.02**	− 2.6 (− 3.5;-2.2)	−2.4 (− 2.6;− 2.2)	0.23	-2.2 (− 2.5;-2.1)	−2.5 (− 2.9;-2.2)	**0.04**
*Weight (SDS)*	−2.4 (− 2.8;-1.7)	− 1.6 (− 2.4;-0.8)	**< 0.01**	−2.4 (− 2.8;-1.4)	−2.5 (− 3.3;-1.8)	0.27	−2.5 (− 3.0;-1.4)	−2.4 (− 2.8;-1.8)	0.77	−2.4 (− 2.8;-1.7)	−2.4 (− 2.9;-1.7)	0.98
*BMI (SDS)*	−1.1 (− 1.9;-0.3)	−1.0 (− 1.6;0.2)	0.59	−1.0 (− 1.9;-0.4)	−1.1 (− 2.0;0.2)	0.62	−0.7 (− 1.7;0.3)	−1.2 (− 2.0;-0.3)	0.20	−1.7 (− 2.2;-0.8)	−1.0 (− 1.7;0.2)	0.20
*Age (years)*	9.1 (6.2;12.4)	12.1 (5.4;14.3)	0.21	11.2 (7.3;13.0)	8.3 (3.7;12.1)	0.82	8.2 (5.3;10.8)	10.2 (7.1;12.6)	0.06	11.3 (10.3;13.0)	8.8 (6.0;12.1)	0.24
*Female (%)*	42%	41%	1.00	33%	50%	0.38	42%	42%	1.00	83%	50%	**0.04**
*Pubertal (%)*	19%	59%	**< 0.01**	21%	21%	1.00	0%	25%	0.09	25%	17%	0.67

*AD-SS* Suspected autosomal dominant short stature, *SS* Short stature, *C-FSS* Constitutional familial short stature, *TH-SS* Target height-related short stature

**Fig. 1:**
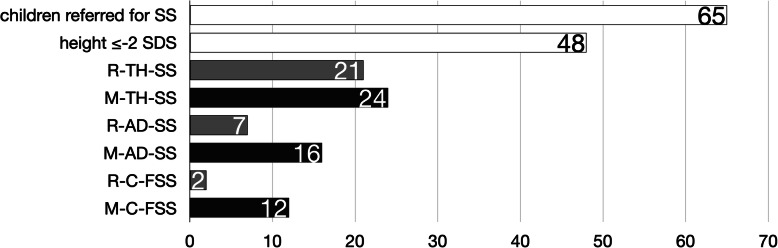
Distribution of children referred for short stature (SS) based on FSS definition and source of parents’ height data (R for referred, in grey; M for measured, in black; TH-SS, target height-related familial short stature; AD-SS, suspected autosomal dominant familial short stature; C-FSS, constitutional familial short stature)

**Fig. 2:**
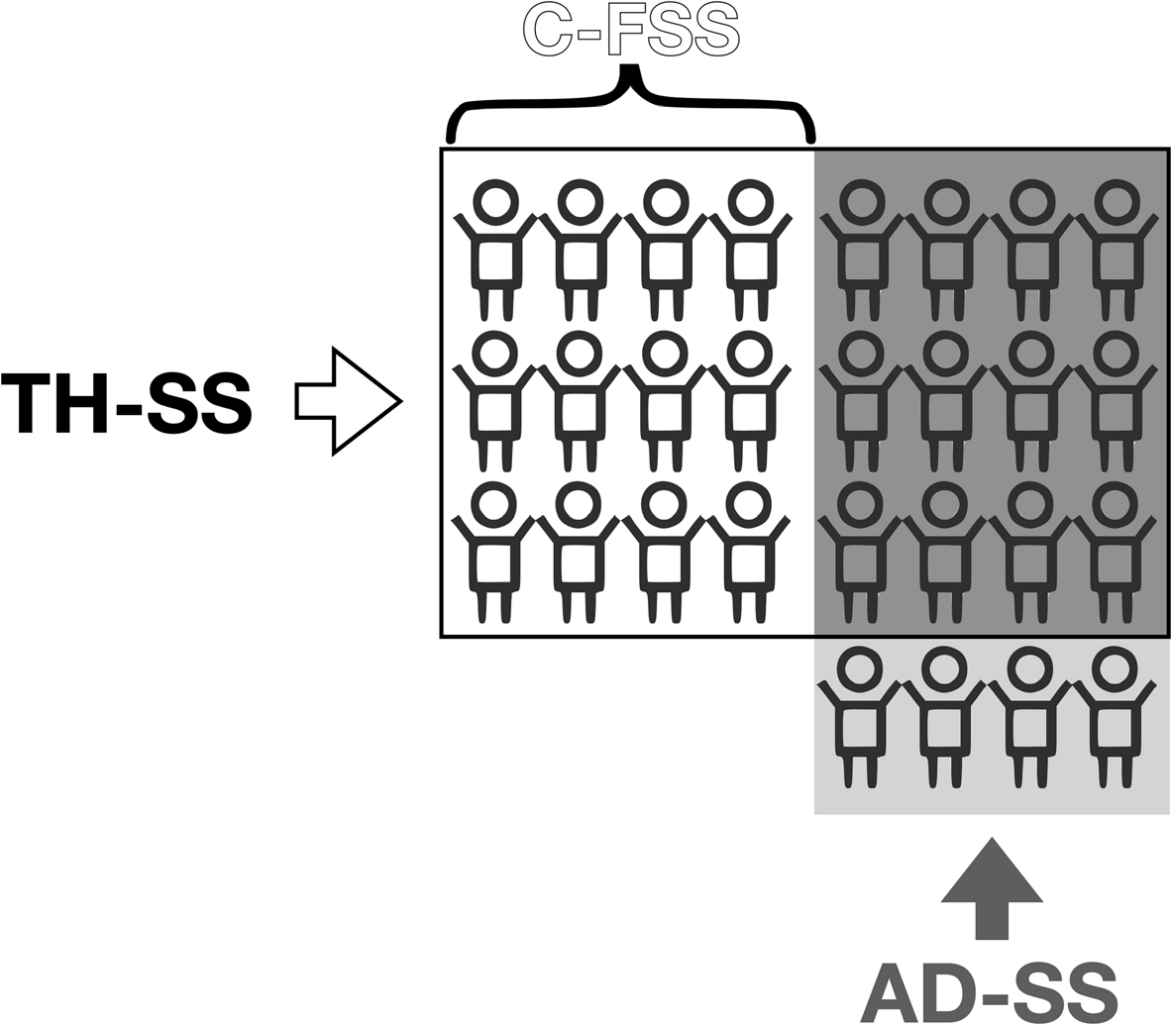
Distribution of children with TH-FSS, AD-FSS, and C-FSS based on measured parents’ heights (TH-SS, target height-related familial short stature; AD-SS, suspected autosomal dominant familial short stature; C-FSS, constitutional familial short stature)

When considering M-PHt, 24 children had TH-SS and 16 children AD-SS (Fig. 1). Overall, 28 children were included in at least one of the two definitions: 12 children were identified by both TH-SS and AD-SS, 12 subjects only by TH-SS (since they did not have any of parents with SS) and represented C-FSS, and 4 individuals were identified only by AD-SS (because their TH was not ≤ − 2 SDS) (Fig. 2)*.*

Median height SDS in children with TH-SS and C-FSS was significantly higher than in children without TH-SS or C-FSS, respectively, while it was lower in children with AD-SS than in children without AD-SS, with no statistical significance (Table 1).

If we had considered R-PHt, 3 out of 24 children with TH-SS, 9 out of 16 with AD-SS, and 10 out of 12 C-FSS would have been missed (Fig. 1).

## Discussion

In this study, we verified that the prevalence of FSS among short children might vary according to novel proposed definitions: 50% if we consider “target height-related short stature” (TH-SS), 33% for suspected “autosomal dominant short stature” (AD-SS) and 25% for “constitutional familial short stature” (C-FSS). Moreover, we found that a relevant quote of FSS may be missed if clinicians only rely on reported parents’ height (56% of AD-SS, 13% of TH-SS, 83% of C-FSS).

The calculation of TH is a standard procedure for every pediatrician over the past 50 years [15]: since 90% of children’s height is known to be within 1.5 SDS (approximately 2 centile lines) of TH [16], if the estimated final height is outside this range, it should be considered a variant growth pattern or a pathologic cause. When a child is short and height is consistent with TH, this has been classically addressed as FSS and considered most often a normal variant [2, 3].

However, TH is based on the assumption of an equal magnitude of polygenic factors derived from both parents, and if one of the parents is unusually tall or short, the TH is a poor predictor of attained height, since genetics is not just a matter of average and the child will inherit traits relating to stature more from one parent than the other [17]. This matter is essential when examining FSS: the presence of at least one parent with a height of ≤ − 2 SDS (which we suggest addressing as AD-SS) should always take priority in the diagnostic approach; otherwise, a potential inherited monogenic condition in an autosomal-dominant pattern can be overlooked and classified as a standard variant [6]. Cases of AD-SS should not be disregarded, as the identification of a causative gene can support treatment decisions (e.g., a more accurate prediction of the specific response to growth hormone treatment), the evaluation of the recurrence risk in the family, and enables the recognition of other features in case of a syndrome [18]. In our cohort, one-third of children with SS could be classified as AD-SS, and among them, three quarters could have been missed if the attention was only pointed on TH.

We suggest considering as “constitutional familial short stature” (C-FSS) only children with TH-FSS that do not have any parents with SS (TH-SS without AD-SS). This subgroup can be considered a normal variant, undeserving further evaluation, after the most frequent causes of short statures, such as coeliac disease or thyroid disorders, have been excluded and statural growth has not presented any further deflections over time.

Moreover, our data underline that heights (of both children and parents) must always be measured directly for usable data. This procedure is necessary for all children referred for short stature, and our data showed that 26% of respondents for this issue were not truly short. However, parents should always be measured, as when they report their height, an incorrect measurement (even higher than 8 cm) is often communicated. In particular, adults of short stature or parents of children with this problem tend to overestimate their height, making TH unreliable [11, 19]. This overestimation has its clinical effects in that reported rather than measured parental heights could have led to losing AD-SS in more than half of our cohort cases.

We then suggest the following workflow in cases of suspected FSS:

1. children’s height must always be measured carefully to confirm SS;
2. the most frequent causes of SS should be ruled out first (such as celiac disease or thyroid disorders) before considering FSS as an etiology, as FSS always remains a diagnosis by exclusion;
3. both parents should always be measured, and their heights should be considered together (to calculate TH) and individually (to exclude parental SS). In practical terms, it is possible to identify a cut-off height for the definition of SS in parents, reducing the need for additional calculations during the medical evaluation (for instance, in the Italian population, − 2 SDS height in adults corresponds to 150.9 cm for women and 164.1 cm for men) [12].
4. if at least one of the parent is ≤ − 2 SDS (AD-SS), a genetic evaluation should be requested in order to investigate genetic mutation causing possible SS with autosomal-dominant inheritance;
5. if a child has a short stature consistent with TH (TH-SS) and none of the parents’ height is ≤ − 2 SDS, we could define it as having C-FSS, intended as a standard variant of growth, with no need of specific further investigation.

Although this study refers to a small cohort in a single-center, we believe that it focuses on the accurate definition of FSS for its following management.

Further studies are needed in order to understand in how many cases of AD-SS a causative gene mutation can be found with newer approaches (e.g., next-generation sequencing or clinical exome sequencing) [4] and in which cases a treatment (e.g., growth hormone or vosoritide) can allow to restoring an average height [20, 21].

## Conclusion

In conclusion, we suggest novel definitions to adequately detect and approach the cases of FSS since C-FSS might not need any specific investigation, while on the contrary, AD-SS should undergo genetic evaluation. Moreover, this study underlines that adequate measurement and consideration of children’s and parents’ heights (individually and together) are crucial in the clinical evaluation of every children with SS.

## Data Availability

The data that support the findings of this study are available from the corresponding author, GT, upon reasonable request.

## Abbreviations

AD-SS: Suspected autosomal dominant short stature
C-FSS: Constitutional familial short stature
CHt: Child height
FSS: Familial short stature
IQR: Interquartile range
M-: Measured
PHt: Parent height
R-: Reported
SDS: Standard deviation score
SS: Short stature
TH: Target height
TH-SS: Target height-related short stature
